# Meckel’s “Wallet”: A Unique Case of Intestinal Obstruction

**DOI:** 10.7759/cureus.64920

**Published:** 2024-07-19

**Authors:** Wila-e-Zehra Syeda, Syeda Amna Azim, Nasir Z Ahmad

**Affiliations:** 1 Colorectal Surgery, University Hospital Limerick, Limerick, IRL

**Keywords:** small bowel, foreign body, coins, meckel's diverticulum, bowel obstruction

## Abstract

While bowel obstruction is the most common surgical disorder of the small intestine, small bowel obstruction due to Meckel's diverticulum is a relatively rare occurrence. We encountered a compelling case of small bowel obstruction that turned out to be more complex than anticipated, involving a Meckel's diverticulum with some unforeseen findings. We followed standard guidelines for history-taking, examination, investigations, and management of the intestinal obstruction. After exhausting conservative treatment options, we opted for surgical intervention, and the unexpected cause of the obstruction took us by surprise. This case report highlights an exceedingly rare entity: Meckel's diverticulum precipitating uncommon complications.

## Introduction

Bowel obstruction basically refers to the failure of intestinal contents to progress forward. It is the most common cause of acute abdomen, accounting for as many as 80% of the admissions due to acute abdomen [1]. It also carries a significant burden of morbidity and mortality [2]. Small bowel obstruction is three to four times more common than large bowel obstruction [2]. There are two categories of small bowel obstruction: (1) functional, with no discernable physical blockage, and (2) mechanical, characterized by a physical blockage hindering the movement of intestinal contents. In this report, we present a rare case of small bowel obstruction associated with Meckel's diverticulum.

## Case presentation

A 61-year-old male with a history of learning disability, psychiatric disorder, and schizophrenia was admitted to the hospital with lower abdominal pain, nausea, and vomiting. He was unable to communicate verbally, but it was reported that he had swallowed a mouthful of coins before arriving at the hospital. There was no history of ingesting sharp objects or chemicals such as lead batteries. Upon examination, his abdomen was slightly distended, and there was no specific tenderness or guarding. Basic hematological testing revealed a typical profile with a hemoglobin count of 13.5 g/dL and a WBC count of 8.2 × 10^9^/L. However, the renal profile indicated evidence of acute kidney injury with a creatinine level of 334 umol/L.

**Figure 1:**
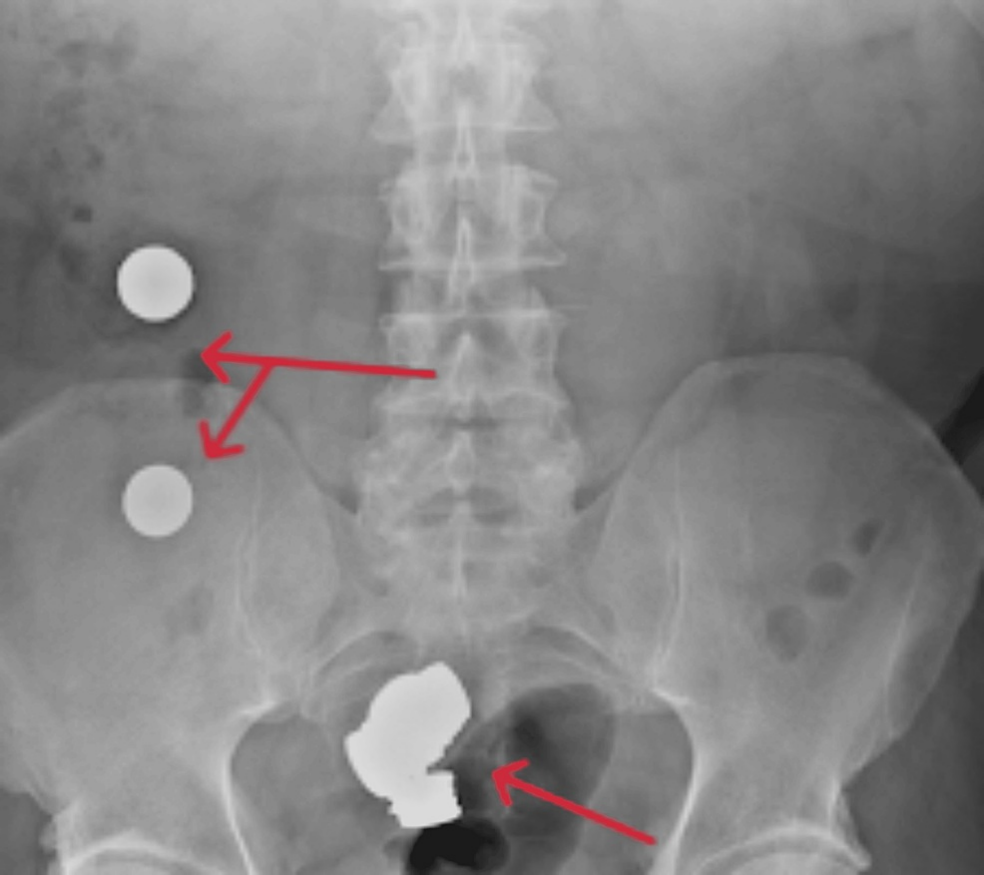
X-ray revealing multiple foreign bodies in the small bowel and cecum (arrows)

**Figure 2:**
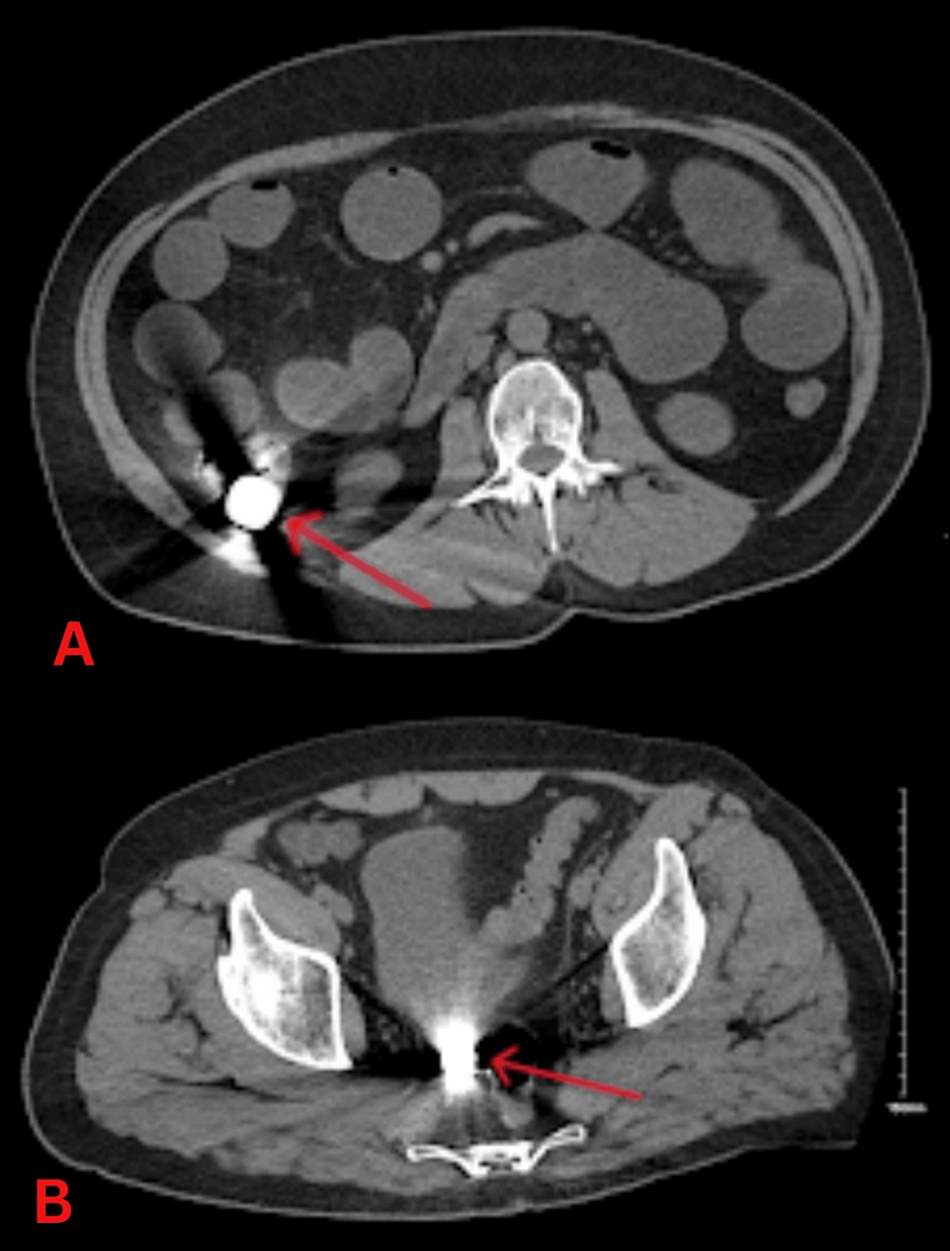
CT scan revealing multiple foreign bodies in the cecum (A) and small bowel (B) (arrows) CT: computed tomography

The coagulation profile also showed some abnormalities with a PT of 15.9 seconds, APTT of 53.0 seconds, and a fibrinogen level of 6.2 g/L. Also, the patient's creatinine kinase level was very high at 2320 U/L. Other test results included a C-reactive protein level of 192 mg/L and a lactate level of 1.6 mmol/L. Serum and urinary amylase were within the normal range. Urine microscopy did not indicate any signs of infection. Furthermore, all viral markers and coronavirus disease 2019 (COVID-19) tests were negative. An abdominal X-ray (Figure 1) revealed the presence of multiple foreign bodies in the small and large bowels, causing a small bowel obstruction without evidence of perforation. These findings were later confirmed by a CT scan of the abdomen and pelvis (Figure 2). Apart from the small bowel obstruction due to ingested coins, no other abnormalities were detected.

Treatment

This was a subacute type of small bowel obstruction, believed to be caused by ingested foreign bodies. Therefore, our initial approach involved managing the patient conservatively for 24 hours via fasting, providing intravenous fluids, and adequate pain relief. A nasogastric tube was inserted for intestinal decompression, and a urinary catheter was used to monitor intake and output. Follow-up imaging revealed foreign bodies in the cecum, ascending colon, and possibly rectum, as well as distention of the proximal small bowel loops consistent with small bowel obstruction. Despite conservative management and measures such as the insertion of peripherally inserted central venous catheters (PICC line) for total parenteral nutrition (TPN) and "drip and suck" techniques, the patient did not show any improvement. The opacities seen on the X-rays did not migrate distally and, eventually, we decided to perform an exploratory laparotomy to address the persistent obstruction surgically.

**Figure 3:**
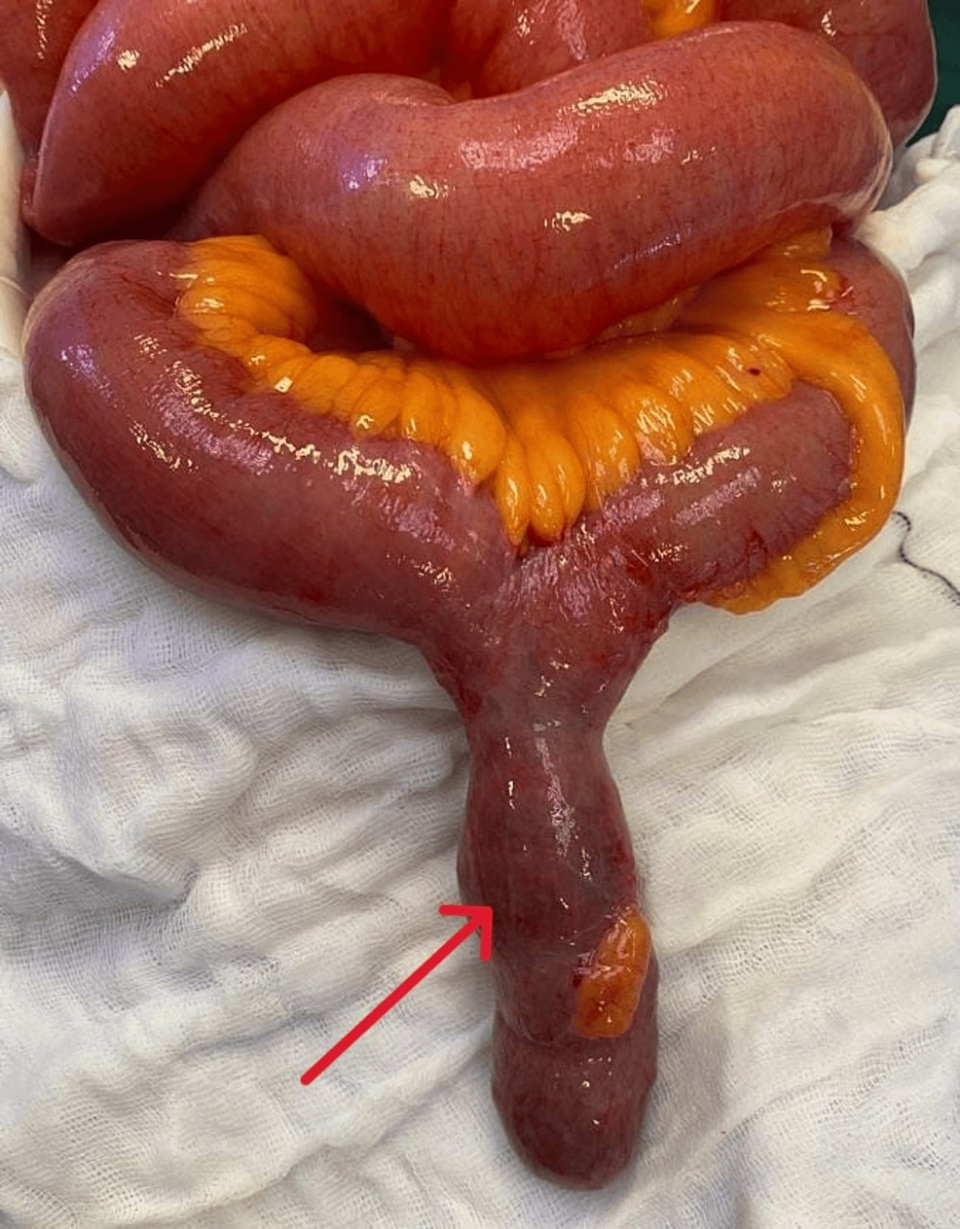
Meckel's diverticulum full of coins (arrow)

During the surgery, it was observed that the small bowel loops were dilated due to an obstruction. The distal ileum and the right and left sides of the colon had collapsed. Surprisingly, the cause of the obstruction was found to be a previously unknown Meckel's diverticulum, which was filled with coins (Figure 3). The diverticulum had expanded because of the presence of coins, and its color was slightly dusky, but there was no sign of perforation. It was hanging into the central pelvis due to gravity, which caused acute angulation and led to the obstruction of the small bowel.

A surgical procedure, Meckel's diverticulectomy with a GIA 80, was performed horizontally, and the staple line was buried with 3/0 PDS. The cecum was also discovered to be filled with tangible coins, but no action was taken to remove them. The surgical incision was closed in the usual manner.

Outcome

Following the surgery, the patient was closely observed. His abdominal distension gradually improved over the next few days, and he was able to start eating solid food. He was discharged from the hospital on the 16th day of his stay, once it was confirmed that he could tolerate a solid diet and was able to pass stool on his own. His caregivers received instructions on how to prevent a similar occurrence in the future. He had a follow-up appointment at the outpatient department (OPD), and no post-surgery issues were identified. Subsequently, he was discharged back to primary care.

The analysis of the tissue showed no signs of cancer or abnormal tissue growth like gastric or pancreatic heterotopia and confirmed that the removed specimen was a Meckel's diverticulum. The detailed examination of the removed bowel tissue further supported the earlier diagnosis of an unidentified Meckel's diverticulum.

## Discussion

Small bowel obstruction can result from various causes, such as adhesions, volvulus, intussusception, hernias, tumors, and occasionally from a bezoar or other undigested foreign body [3]. Among all the causes of intestinal obstruction, a Meckel's diverticulum and its complications are considered relatively rare [4]. Due to its rarity, lack of specific symptoms, and similarity of presentation with other surgical emergencies, we may not include it in the differentials in a case of small bowel obstruction, which happened in our case too. Traditionally, small bowel obstruction was treated with early operative measures, as evident by the century-old proverb, “Never let the sun rise or set on a bowel obstruction”. It was considered the best modality for ensuring minimal morbidity and mortality. In the 1930s, conservative management and nasogastric decompression were established as an alternative option with the potential of saving patients from laparotomy [5], leading to the emergence of modern-day protocols for small bowel obstruction.

Conservative management involves methods like NGT decompression, NPO diet, electrolyte management, and close monitoring. This approach is recommended as the initial treatment for uncomplicated adhesive small bowel obstruction [5]. As our working diagnosis was intestinal obstruction secondary to ingested coins, we continued with conservative measures anticipating that coins would eventually pass. Generally, if there are any signs or symptoms of bowel compromise or if the patient's condition does not improve with conservative management after three to five days, operative management is necessary [5]. When the serial X-rays in our case did not show any migration of the coins, we decided to operate to prevent the risk of bowel perforation.

As a standard practice, operative management could be the first choice of treatment if the cause of small bowel obstruction is unlikely to be resolved with conservative management, or if there is an immediate risk of bowel ischemia [5]. In our case, a Meckel's diverticulum along with the ingested foreign bodies was responsible for intestinal obstruction. The other mechanisms of obstruction due to a Meckel's diverticulum include volvulus, intussusception, Littre's hernia, axial torsion, stricture, lithiasis, tumors, mesodiverticular band, and entrapment of small bowel beneath a band extending from Meckel's diverticulum to the base of mesentery [6].

The appropriate duration for conservative management varies from case to case and is still a matter of debate. In our case, we closely monitored the passage of coins through the gastrointestinal tract through X-rays and expected that the coins would pass naturally without any intervention. Certain narrow areas within the gastrointestinal tract can cause foreign body impaction. These areas include the upper and lower esophageal sphincter, pylorus, ligament of Treitz, and ileocecal valve [7]. It is believed that once the foreign body has passed through about 800 cm, it is likely to be safely expelled through the anus [7]. In this case, the coins passed through the ileocecal valve were left alone in the cecum and ascending colon and observed without further intervention. Eventually, they passed through the feces without complications.

The impaction of foreign bodies within the Meckel’s diverticulum is somewhat uncommon, and the impingement of such a large number of coins causing a pull because of the weight of coins and leading to obstruction is even rarer. A Meckel's diverticulum is the most common congenital gastrointestinal anomaly. It is considered a true diverticulum because it contains all three layers of the small intestine alongside potential ectopic gastric mucosa. During embryogenesis, if the omphalomesenteric duct is partially obliterated and persists, it gives rise to Meckel's diverticulum. Meckel's diverticulum is mainly an incidental finding on imaging or during surgery; only a few cases ever become symptomatic [4]. It affects 2% of the population [8]. Most cases remain asymptomatic throughout life, with only about 4% needing hospital admission and only about 3% requiring surgical intervention. The most common complications are bleeding, diverticulitis, and obstruction. It can also present as an inguinal hernia (Littre’s hernia), but this is less frequent [9].

## Conclusions

We discussed a rare case of intestinal obstruction where two different mechanisms contributed to the clinical picture. The presence of foreign bodies in the gastrointestinal tract was evident from the history and X-rays, but an element of impaction halted the passage of coins distally. The factor responsible for the impingement of coins was Meckel’s diverticulum, which was completely concealed and not detected in the preoperative imaging. Furthermore, the large number of coins within the diverticulum halted their migration rather than the Meckel’s itself. Intestinal obstruction is a common cause of surgical admissions and often requires laparotomy if conservative measures do not improve the clinical condition. In our case, in the surgeon’s opinion, the role of laparoscopy in intestinal obstruction was limited because the distension of the bowel restricts the safe manipulation with laparoscopic instruments. The presence of multiple foreign bodies in Meckel’s diverticulum is a rare occurrence, but it is important to consider this likelihood when dealing with intestinal obstructions.
